# Histopathological and Molecular Characteristics of Pediatric Adrenocortical Tumors: where Do we Stand?

**DOI:** 10.1007/s12022-026-09905-3

**Published:** 2026-02-19

**Authors:** Christianne C. Swart, Ronald R. de Krijger

**Affiliations:** 1 Princess Máxima Center for Pediatric Oncology, Utrecht, The Netherlands; 2 Department of Pathology, University Medical Center Utrecht, Utrecht, The Netherlands

**Keywords:** Pediatric adrenocortical carcinoma, Histopathological scoring systems, Molecular classification, Cancer predisposition syndromes

## Abstract

Pediatric adrenal cortical carcinoma (ACC) is a rare heterogeneous cancer type that is incompletely understood and differs from its adult counterpart in clinical presentation, histopathological characteristics, genomic landscape, and prognosis. Pediatric ACC has a bimodal age distribution for children with ACC, with the highest incidence in young children and adolescents. Tumors commonly present with an endocrine syndrome such as virilization or Cushing’s syndrome, associated with hormone overproduction. Surgical resection is the primary treatment. Adjuvant therapies such as mitotane or chemotherapy can lead to (severe) side effects in children and should be closely monitored. Histopathological assessment commonly relies on the AFIP/Wieneke classification, with the modified reticulin algorithm also providing predictive value. Recently, the two-step scoring system by Picard aimed to integrate the AFIP/Wieneke classification and the Children’s Oncology Group (COG) staging system into a two-step model. Molecularly, pediatric ACC is strongly associated with germline variants of *TP53* and loss of heterozygosity of chromosomes 11 and 17. Somatic variants in several genes, including *ATRX* and *CTNNB1,* have been identified and are associated with poor prognosis. Multiple factors, such as age, tumor size, and biomarkers, with Ki67-labeling index being most important, are of prognostic value. Despite research advances, overall survival remains poor and worsens with older age at diagnosis and advanced disease. To improve survival rates of pediatric ACC further research is necessary aiming at optimizing therapeutic strategies in these patients. This review summarizes current knowledge of this challenging tumor and highlights recent advances in the field.

## Introduction

Adrenocortical carcinoma (ACC) is a very rare tumor type with an annual incidence of 1.02 per million people in the United States [1]. Despite its rarity, ACC is the most common primary adrenal malignancy in adults [2]. In children, ACC is uncommon, with an annual incidence of 0.21 per million people annually [3]. The age distribution in patients with ACC shows two peaks; before the age of 4 years and a peak between 40 and 50 years, with an overall higher incidence in females [4, 5]. Interestingly, research shows that there are clear distinctions between pediatric and adult ACC at both the molecular and histological level, which should be taken into account when diagnosing and treating these tumors [6].

Pediatric ACCs are strongly associated with cancer predisposition syndromes (CPS), including Li-Fraumeni syndrome (LFS) and Beckwith-Wiedemann syndrome (BWS) [7], with the majority of pediatric ACC cases involving germline variants that drive early-onset tumorigenesis [8, 9]. For example, in the Brazilian population, a founder germline *TP53* variant causes a higher incidence of ACC in children [10–12]. To predict clinical outcomes in adult ACC, the Weiss scoring system was developed based on histopathological characteristics [13]. As this scoring system does not suffice for pediatric ACC, the AFIP/Wieneke score was developed at the beginning of this century to distinguish malignant from benign pediatric adrenal cortical tumors (ACT). However, there is no clear-cut correlation between histopathological classification and clinical outcome. ACTs lacking malignant features generally exhibit benign behavior. In contrast, tumors within the intermediate range of AFIP/Wieneke score may be associated with either a good or a poor outcome. This variability complicates prognostic assessment in pediatric ACTs and should therefore be addressed [14]. Clinical presentation of children with ACC differs from that of adults, as children are more likely to present with functional tumors that secrete hormones, leading to symptoms such as virilization and Cushing’s syndrome [15].

The 3-year overall survival (OS) rate for children with ACC is approximately 55% [15, 16]. Although surgery is the cornerstone of treatment for pediatric ACC, significant knowledge gaps remain regarding other therapeutic options, which are largely extrapolated from adult ACC treatment strategies. For example, mitotane or chemotherapy are both used to treat adult ACC, but are also applied to pediatric ACC, as studies show higher OS rates using these adjuvant therapies in adults [15, 17]. In children, these adjuvant treatments can cause severe side effects, such as neurotoxicity and/or precocious puberty, and should be closely monitored [18].

As discussed, pediatric ACC should be considered a distinct tumor entity from the adult variant. However, its etiology and pathogenesis remain incompletely understood. Greater insight into pediatric ACC biology may improve prognostic accuracy and might offer new therapeutic perspectives. This review aims to describe the histopathological and molecular characteristics of pediatric ACC.

## Histopathology

Currently, in adult ACTs, different scoring systems are used, including the Weiss score, modified Weiss score, Helsinki score, reticulin algorithm, and the Lin-Weiss-Bisceglia system [19–22]. Different studies show that most of these systems cannot be used to classify pediatric ACTs because of their distinct histopathology [14, 23]. The Weiss score tends to overestimate the malignant potential of pediatric ACTs, resulting in overdiagnosis of ACC [23]. In 2003, AFIP/Wieneke et al. proposed a classification specifically for pediatric ACT, followed by Picard et al. in 2019, proposing a two-step model [14, 24].

### Scoring of Adult Adrenocortical Tumors

The Weiss score consists of 9 histologic features (see Table 1), indicating malignancy if three or more features are present. A low mitotic rate (<20 mitotic figures per 10 mm^2^) and absence of necrosis have been found to be strong predictors of OS, whereas other factors were not linked to patient outcome [13, 19, 25]. Although the Weiss score has been shown to accurately identify malignancy, the modified Weiss score was subsequently developed based on the most relevant criteria: mitotic rate, the presence of clear tumor cells, abnormal mitosis, necrosis, and capsular invasion [26]. In addition, the reticulin algorithm was designed, focusing on reticulin framework disruption and analyzing mitoses (>5 mitoses per 10 mm^2^), necrosis, and venous invasion are analyzed. If a disrupted reticulin framework is accompanied by one of these factors, the tumor is classified as an ACC. This score is easier to apply than the Weiss score, as it excludes subjective parameters such as sinusoidal invasion or nuclear atypia [21]. The Helsinki score can predict the metastatic probability of ACTs by including mitotic rate, necrosis, and proliferation rate of the tumor. Mitotic rate and necrosis are stronger predictors and are therefore weighed in the calculation, which is performed as follows: 3 x mitotic rate + 5 x necrosis + proliferation index (PI). PI is determined using Ki67 staining. An ACT score above 8.5 points is considered malignant, and a score above 17 is related to worse survival, making the Helsinki score both a diagnostic and prognostic tool [20]. The Lin-Weiss-Bisceglia system is developed specifically for oncocytic adrenocortical tumors and defines three groups, based on major criteria (high mitotic rate, atypical mitoses, or venous invasion) and minor criteria (large size, necrosis, or capsular invasion). If none of these criteria are present, the oncocytic tumor is classified as benign. Tumors exhibiting one or more minor criteria are classified as borderline, whereas the presence of any major criterion defines the tumor as malignant [22]. In conclusion, many studies have been done defining which parameters are most related to malignancy, metastasis, or outcome. Mitotic rate and necrosis appear to be key features of ACC, but it remains challenging to design the ideal scoring system, and further research is needed to refine existing scoring systems.

**Table 1:** Comparison between AFIP/Wieneke criteria [14] and Weiss score [19], adapted from original publications

AFIP/Wieneke criteria	Weiss score
**Comparable features**	
Venous invasion	Invasion of venous structures
Capsular invasion	Invasion of tumor capsule
Presence of tumor necrosis	Necrosis
>15 mitoses per 4 mm^2^	Mitotic rate: >5 mitoses per 10 mm^2^
Presence of atypical mitotic figures	Atypical mitosis
**Distinct features**	
Tumor weight > 400 g	Nuclear grade III or IV
Tumor size >10.5 cm	Clear cells comprising 25% or less of the tumor
Extension into periadrenal soft tissues and/or adjacent organs	Diffuse architecture
Invasion into vena cava	Invasion of sinusoidal structures
**Total score**	
0–2: benign 3: indeterminate for malignancy ≥ 4: poor clinical outcome	≤ 2: most likely benign ≥ 3: malignant

### Scoring Pediatric Adrenocortical Tumors

#### AFIP/Wieneke Classification

**Fig. 1:**
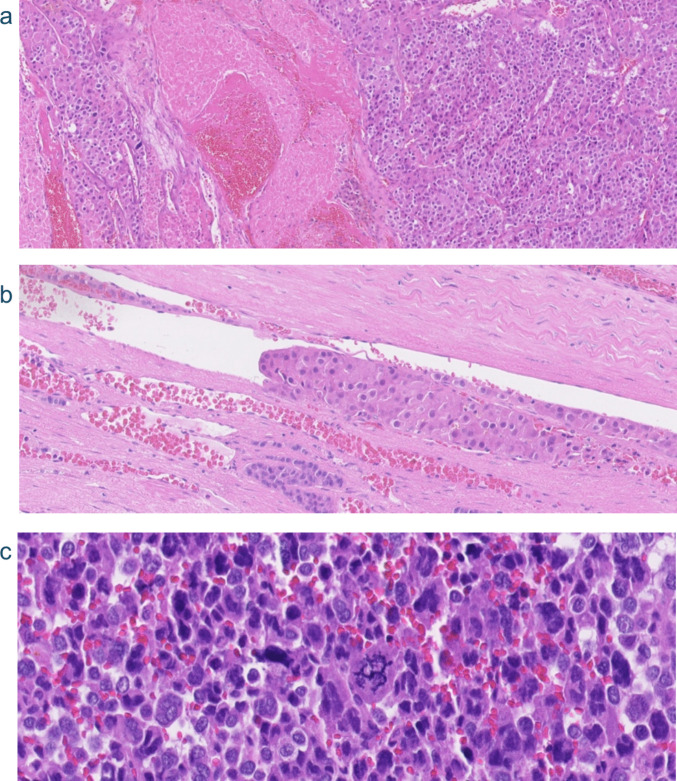
Hematoxylin and eosin (H&E)-stained sections showing morphological features of pediatric adrenal cortical carcinoma. A and B come from a sporadic ACC in a 15-year-old girl and show necrosis and venous invasion respectively. C comes from an ACC in a 5-year-old boy with Li-Fraumeni syndrome and shows profound nuclear pleomorphism and an atypical mitotic figure

The AFIP/Wieneke classification is the most frequently used scoring system for pediatric ACTs. Some criteria of the Weiss score are also incorporated into the AFIP/Wieneke score, but with different cut-off values. A comparison of similarities and differences between AFIP/Wieneke and Weiss classification is displayed in Table 1. Tumor weight > 400 g and size > 10,5 cm are macroscopic features included in the AFIP/Wieneke classification. However, neither weight nor size can be used as an independent predictive factor of outcome. Extension into periadrenal soft tissue and/or vena cava invasion are macroscopically and microscopically identifiable features. In rare instances, these features have been described in clinically benign but, pathologically malignant ACT [11]. However, in most cases these features are associated with clinically and pathologically malignant ACT and vena cava invasion is an independent predictor of outcome [14]. The microscopic features of the AFIP/Wieneke classification include venous invasion (Fig. 1b), capsular invasion, tumor necrosis (Fig. 1a), increased mitotic activity (>15 mitoses per 4 mm^2^) and the presence of atypical mitotic figures (Fig. 1c). None of these characteristics is individually pathognomonic for poor outcome in ACC and therefore cannot be used alone for prognostication. Follow-up studies are not concordant regarding the importance of the microscopic features. Das et al. (2016) found capsular invasion and venous invasion in 6 out of 7 ACCs but not in adrenocortical adenoma’s (ACAs), suggesting high specificity of these criteria. High mitotic rate (>15 mitoses per 4 mm^2^) was seen in all ACCs but also in two ACAs [27]. Three groups can be identified based on the AFIP/Wieneke criteria: benign (score ≤ 2), indeterminate for malignancy (score 3) and poor clinical outcome (score ≥ 4). Seventy-eight per cent of the cases in this study could be correctly categorized using these cut-offs [14].

The AFIP/Wieneke classification has been compared to multiple scoring systems. A study comparing the accuracy of several classification systems in pediatric ACC showed the highest accuracy of 77% using the AFIP/Wieneke criteria to classify pediatric ACT [28]. Another study containing 24 children suggested using AFIP/Wieneke criteria as the gold standard, since it correctly matched clinically and pathologically benign or malignant tumors [29]. AFIP/Wieneke classification has also been compared to the reticulin algorithm and Helsinki score. In 26 children with ACT, the Helsinki score was most specific and accurate (respectively 81.25% and 80.77%), and the reticulin algorithm was most sensitive (100%). Regarding the Helsinki score, a value above 24 was associated with poorer patient outcomes, suggesting this threshold may serve as a cut-off point in pediatric ACTs. Interestingly, in this study, the Helsinki score and reticulin algorithm performed better than the AFIP/AFIP/Wieneke system in the accurate identification of cases with poor outcome [30]. Consequently, the AFIP/Wieneke classification may have limitations in defining malignancy, as it categorizes some patients in the intermediate group, who ultimately experience a benign outcome [24, 30]. This should be taken into consideration when using this classification, as misdiagnosis could lead to unnecessary stress for patients and their family.

#### Modified Reticulin Algorithm

The reticulin algorithm (RA) was originally developed for adult ACT scoring as described in the first paragraph of this chapter. More recently, a modified version of the RA (pRA) was shown to predict outcomes in the pediatric population as well. The disruption of the reticulin framework, when combined with high mitotic rate (>15 mitoses per 4 mm^2^), necrosis, and venous invasion, was associated with poor prognosis. The predictive value of pRA was comparable to the AFIP/Wieneke score, but it is easier to apply, making it a useful diagnostic tool [31].

#### Scoring System Developed by Picard

In 2019, Picard et al. proposed another scoring system for pediatric ACTs to guide treatment decisions by reviewing the AFIP/Wieneke classification. First, they argued that a weight cut-off of 400 g is too high, as tumor weight above 200 g is already associated with malignancy. Moreover, the AFIP/Wieneke score considers size and weight as independent parameters, which may lead to an overestimation of malignancy risk due to the inherent correlation between these variables. Second, noted that vena cava invasion is primarily detected through imaging and therefore does not contribute to histopathologic characterization. The two most important indicators for malignancy in the study by Picard were necrosis and capsular invasion, although necrosis may also occur in benign cases [24]. Due to a lack of concordance in defining atypical mitotic figures between pathologists, this feature is not sufficiently reproducible. In addition to morphology, Ki67 is a useful marker for detecting proliferation in ACTs and a predictor of clinical outcome. The Ki67 labeling index (LI) is determined by counting stained nuclei in hotspot areas with the highest labeling. An index of <15% was found in all benign cases, which makes this cut-off a useful parameter for selecting benign and malignant tumors [24].

**Fig. 2:**
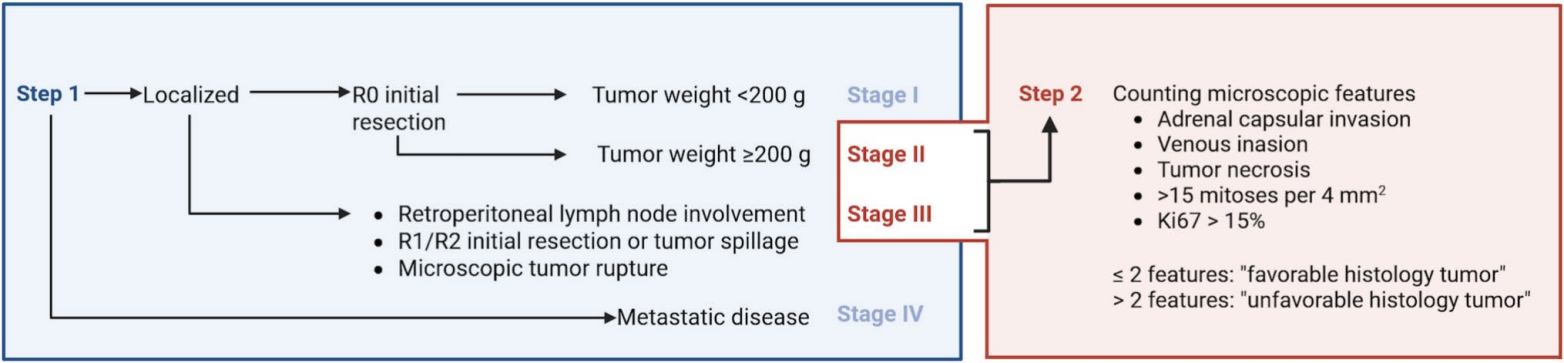
Two-step scoring system developed by Picard et al. [24]. In the first step, the COG stage is defined. R0 = complete surgical resection, R1 = microscopic residual after surgical resection, R2 = macroscopic residual after surgical resection (adapted from Picard et al. (2019)). Created in BioRender. De Krijger, R. (2026) https://BioRender.com/kmi3uhs)

ACTs are also staged, as discussed in the next paragraph. Stage I tumors are mostly treated by surgery alone, while stage IV tumors are generally treated systemically in addition to surgery, regardless of the histologic criteria. In addition to its diagnostic qualities, the AFIP/Wieneke classification helps determine the need for systemic adjuvant treatment in stage II and III tumors. However, Picard et al. demonstrated the redundancy of some of the features of the Children’s Oncology Group (COG) staging and AFIP/Wieneke classification. Hence, tumor weight, size, vena cava invasion, and invasion into adjacent tissue/organs are considered separately from five microscopic factors that are significantly associated with prognosis. This distinction led to a two-step model (Fig. 2), in which staging is first determined based on the COG staging system (Table 2) [32]. Patients with stage II and III disease qualify for step 2. In step 2, a histopathologic score is assigned based on 5 elements, each contributing one point: adrenal capsular invasion, venous invasion, tumor necrosis, >15 mitoses per 4 mm^2,^ and Ki67 > 15%. A score of ≤2 is related to favorable histology, whereas a score >2 is related to unfavorable histology, leading to possible systemic adjuvant therapy [24].

**Table 2:** Staging criteria as defined by UICC/AJCC and ENSAT, Sandrini and COG. UICC/AJCC and ENSAT staging criteria are used for adult ACTs and Sandrini and COG staging criteria are used for pediatric ACTs. The following TNM criteria apply to UICC/AJCC and ENSAT staging only: T1: ≤ 5 cm, no extra-adrenal invasion, T2: > 5 cm, no extra-adrenal invasion, T3: any size, local invasion, T4: any size, invasion in adjacent structures or major vein tumor thrombus, N0: no lymph node metastasis, N1: metastasis in lymph node(s), M0: no distant metastasis, M1: distant metastasis. UICC/AJCC: Union for International Cancer Control and American Joint Committee on Cancer, ENSAT: European Network for the Study of Adrenal Tumors, COG: The Children’s Oncology Group

Stage	UICC/AJCC and ENSAT [33, 34]	SANDRINI [35]	COG [32]
I	T1N0M0	Tumor totally excised, volume < 200 cm^3^, absence of metastasis, normal hormone levels after surgery	Completely resected, small tumors (< 100 g and < 200 cm^3^) with normal postoperative hormone levels
II	T2N0M0	Microscopic residual tumor, tumor > 200 cm^3^, tumor spillage during surgery, or persistence of abnormal hormone levels after surgery	Completely resected, large tumors (≥ 100 g or ≥ 200 cm^3^) with normal postoperative hormone levels
III	T3/4N0M0 T1/2/3/4N1M0	Gross residual or inoperable tumor	Unresectable, gross, or microscopic residual disease Tumor spillage patients with stage I and II tumors who fail to normalize hormone levels after surgery Patients with retroperitoneal lymph node involvement
IV	T1/2/3/4N0/1M1	Distant metastasis	Presence of distant metastases

### Pathological TNM-Stage

Tumor types are mostly classified by the TNM classification, designed by the Union for International Cancer Control and American Joint Committee on Cancer (UICC/AJCC). In the eighth edition [33], the T in ACC is based on tumor size (T1 or T2), the presence of invasion in adjacent structures (T3) or organs or blood vessels (T4). N0 is defined as no lymph node metastasis, and N1 as metastasis in lymph node(s). M0 and M1 are used to distinguish between patients without metastasis and those with distant metastasis, respectively**.** Combining these TNM categories determines the cancer stage (Table 2). The European Network for the Study of Adrenal Tumors (ENSAT) staging system differed from the seventh edition of the UICC/AJCC but was proven to be more accurate. With the advent of the eighth edition, the ENSAT and UICC/AJCC staging systems are identical [33, 34].

Because pediatric ACTs often behave differently in comparison to adult ACTs, a distinct staging system is used. Stage I is assigned to tumors that are completely resected, have a volume of less than 200 cm^3^, show no metastasis, and demonstrate normal hormone levels after resection. Stage II tumors are larger, not completely resected (microscopically), and have deviant hormone levels. Tumor spillage during surgery is also a criterion of stage II. Stage III tumors are not (completely) resectable, and in stage IV, distant metastases are found. Stage I shows the best clinical outcome, with a small tumor (volume < 200 cm^3^), localized disease, and normal hormone levels, while stage IV indicates metastasis, predicting a poor outcome. Stages II and III are less well defined and show a variable outcome [35]. The COG modified the staging system by Sandrini (Table 2), while investigating patient’s outcome after treatment [32]. Approximately half of ACT patients are diagnosed with stage I disease, associated with an estimated 5-year survival of approximately 80%. Survival rates decline to approximately 40% in stage II disease, and to (slightly) above 20% in stages III and IV. Five-year OS is 55% when stage is not taken into account [15, 16]. Because the COG staging system is integrated into Picard’s scoring system, this seems the most practical system to use, but further research is needed to determine the optimal staging system.

## Molecular Background

### Germline Alterations and cancer Predisposition Syndromes

#### The *TP53* R337H Founder Variant in Southern Brazil

Germline *TP53* variants are commonly seen in children with ACC. In these patients, loss of heterozygosity (LOH) of chromosome 17, on which *TP53* is located, is seen. Certain *TP53* variants lead to genomic instability and to multiple other gene variants with the potential to further drive tumorigenesis [6, 36]. Approximately 60% of pediatric patients (age <20 years) with ACC are carriers of a germline *TP53* variant. The presence of germline variants declines with age of diagnosis; approximately 56% of patients with a germline *TP53* variant are aged <4 years [9]. Germline *TP53* variants causing ACC are different from *TP53* variants causing other types of cancer. For instance, more hotspot *TP53* variants are seen in patients with LFS presenting with brain or bone cancer. Furthermore, the penetrance of germline *TP53* variants causing ACC is lower, probably due to residual p53 activity [9].

In southern Brazil, ACTs are more prevalent due to a specific *TP53* founder variant. A variant in codon 337 causes substitution of arginine by histidine (R337H). Almost every child from this region with ACA or ACC carries the R337H variant. LOH was found in most tumors, with the variant allele still present [37]. In 30,000 DNA samples of people from Sao Paulo State, without selecting for disease, 0.21% of the R337H variant was observed. This variant is also correlated to the presence of ACTs [38]. As the presence of the R337H variant is higher in southern Brazil, leading to a higher incidence of ACC in children, screening of families could be cost-effective [39].

The R337H variant most likely originates from one individual, making it a founder variant. Initially, distinct haplotypes were found between patients with the R337H variant, suggesting that the founder effect did not play a role [37]. Later, several studies demonstrated the possibility of a common ancestor, contradicting the findings by Ribeiro et al. (2001). Identical haplotypes were found in 95% of patients with ACTs and the germline R337H variant, suggesting the possibility of a common ancestor for this specific variant [11, 40].

#### Li-Fraumeni Syndrome

**Fig. 3:**
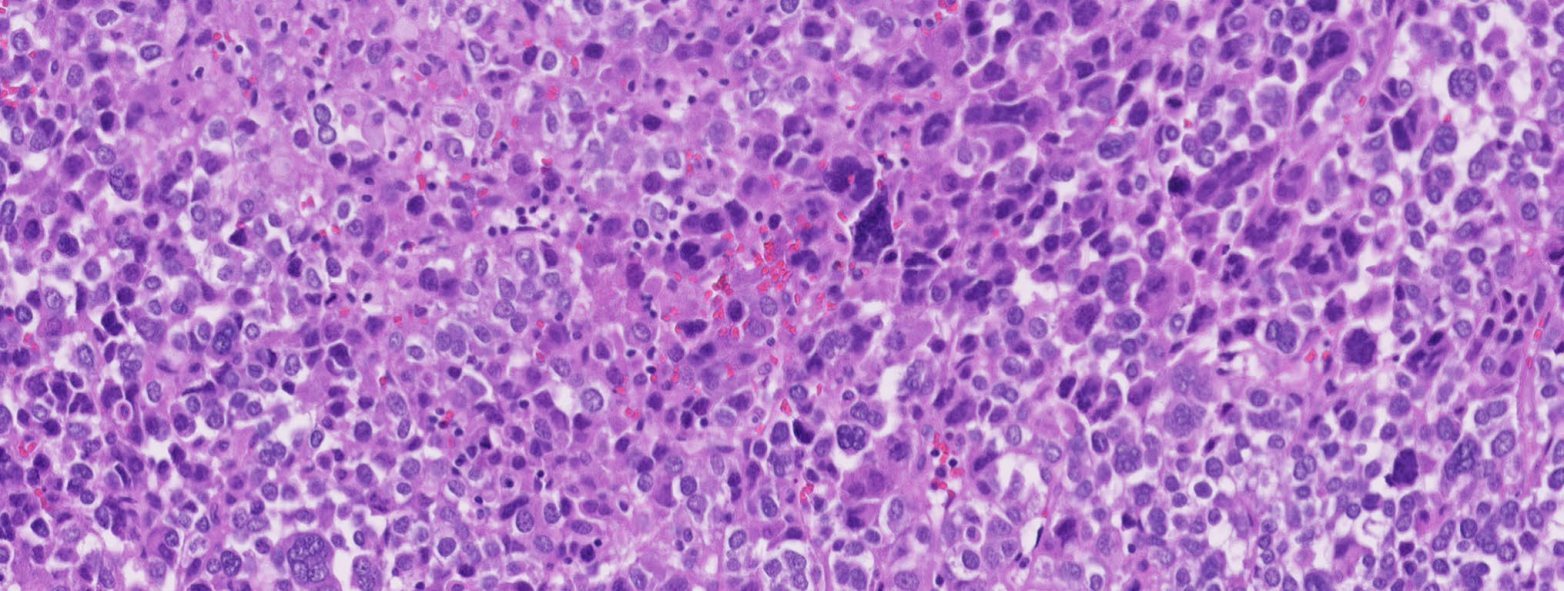
Atypia and anaplasia in an ACC from a 5-year-old boy with LFS

LFS is a CPS associated with multiple cancer types, such as breast cancer, bone and soft tissue sarcomas, brain tumors, and ACC (Fig. 3). The syndrome is inherited in an autosomal dominant manner and caused by germline *TP53* variants, often in the highly conserved DNA-binding domain. [41]. In 265 families with LFS, all patients with adrenal gland tumors (6.5%) were carriers of a *TP53* germline variant and had a median age of 3 years at diagnosis. This highlights the important role of LFS in pediatric ACC, as in patients with sporadic ACC, the median age of diagnosis was 41.9 [42]. Although LFS is caused by variants of *TP53*, the R337H variant in *TP53* has lower and variable penetrance than the other variants causing LFS. In 55 families of patients with ACT with the R337H variant, a significantly higher percentage of cancer (17.6%) was seen in families carrying this variant, compared to non-carriers (6.7%), concluding this variant increases the risk of developing cancer. However, when comparing the family lines with the R337H variant to data of patients with ACT due to a different *TP53* variant, several distinctions were found. Earlier onset was seen in families with a non-R337H *TP53* variant; 76.4% of individuals with cancer were diagnosed before the age of 45. In contrast, only 27.3% of individuals from the R337H family line with cancer were diagnosed before the age of 45. Furthermore, only 4.7% of R337H carriers developed multiple cancers, which is a much lower percentage than in most LFS families. Lastly, the distribution of cancer types differs; gastric, intestinal, and larynx cancers are relatively more present in families of patients with ACT with R337H, compared to breast, bone, and brain cancers in families of patients with ACT, but with a different *TP53* variant [10].

#### Lynch Syndrome

Variants of genes in the DNA mismatch repair system (MMR) are associated with Lynch syndrome; a CPS mostly associated with a higher risk of developing colorectal cancer and endometrial cancer. Adults with Lynch syndrome are also predisposed to developing ACC [43]. In a cohort of 35 children with ACT, 3 were carriers of pathogenic MMR variants, suggesting these variants predispose to the development of pediatric ACC. These children were also carriers of a *TP53* variant [44]. Additional studies are required to assess the potential role of routine genetic counseling for Lynch syndrome in pediatric ACC patients.

#### Beckwith-Wiedemann Syndrome

**Fig. 4:**
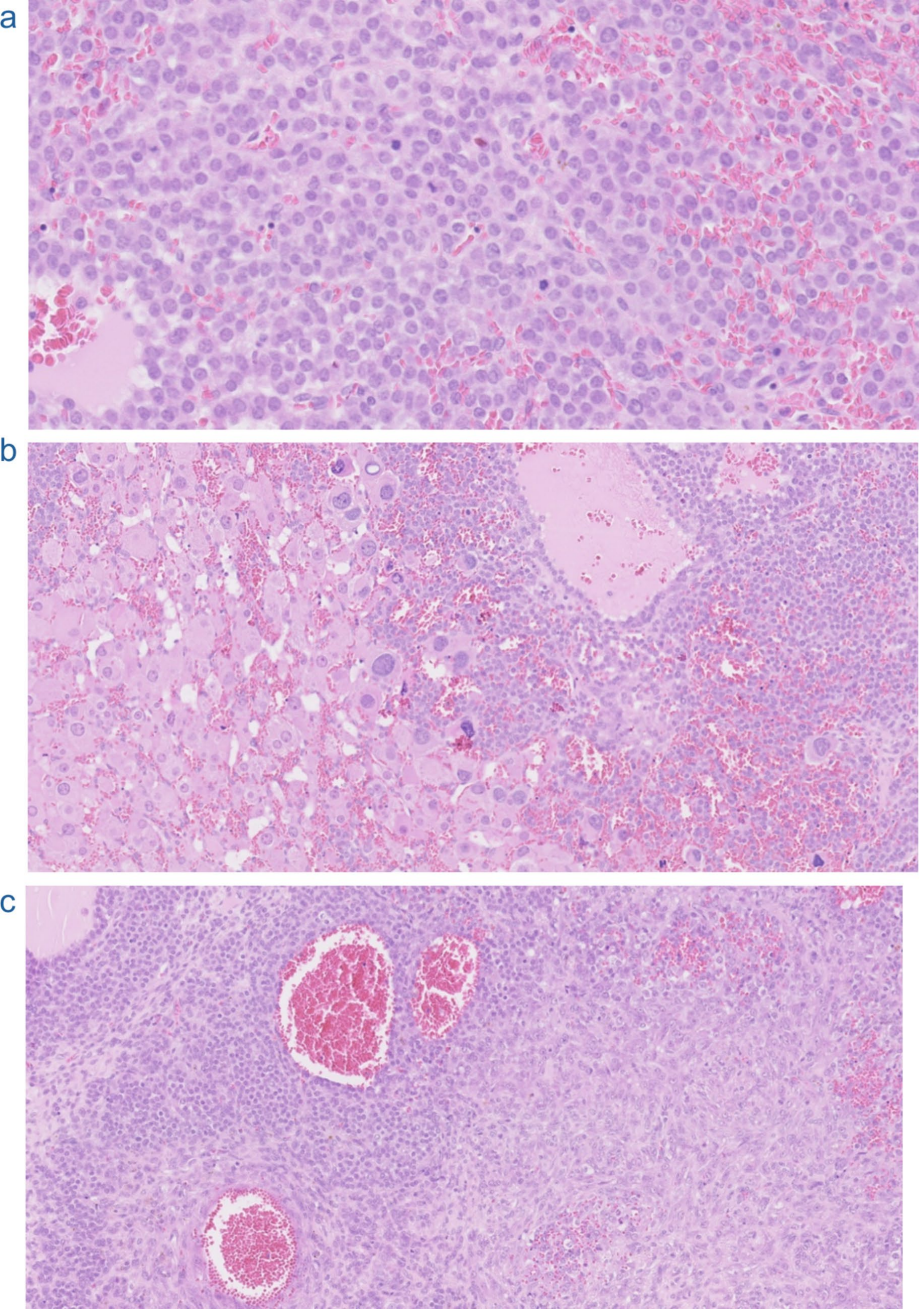
Hematoxylin and eosin stainings of adrenocortical tumor from a 7-year-old girl with Beckwith-Wiedemann syndrome. **A**. Close-up of cellular area featuring numerous mitoses. **B**. Transition between a cellular tumor area to a less cellular area with marked atypia in cells with ample eosinophilic cytoplasm. **C**. Transition of epithelioid cellular area (as in A) to spindle cell area. The tumor cells were stained for alpha-inhibin and Melan A; the Ki67 labelling index was 20%

Beckwith-Wiedemann syndrome (BWS) is a CPS caused by genetic or epigenetic variants of chromosome 11p15. The genes located on this chromosome include *IGF2*, *CDKN1C*, *KCNQ1,* and *H19*, which are associated with growth regulation. In about 50% of patients with BWS, methylation patterns are incomplete, affecting the imprinting center 2 (IC2), which regulates *CDKN1C* and *KCNQ1/KCNQ1OT1* expression. These genes will therefore be differentially expressed. Another epigenetic alteration is the gain of methylation on IC1, leading to the overexpression of IGF2. Genetic alterations include paternal uniparental disomy, translocations, duplications, or inversions of certain genes in the 11p15 region. In approximately 15% of cases, genomic alterations are unknown. Diagnosis of BWS is made clinically through major findings, including abdominal wall defects, macroglossia, and macrosomia, and minor findings, including neonatal hypoglycemia, nevus flammeus, and cardiac abnormalities. Embryonal tumors are often seen in childhood, with Wilms tumor being most frequently observed [45]. ACT is one of the tumor types associated with BWS (Fig. 4). In a review by Carli et al. (2024) about 54 BWS patients, there were 19 with ACA and 32 with ACC, 3 cases being of uncertain malignancy. Patients with ACA presented more frequently with macroglossia and abdominal wall defects, compared to patients with ACC. Thus, patients with ACA scored higher on the Beckwith-Wiedemann Spectrum (BWSp) clinical score, which is based on cardinal and suggestive features such as macroglossia. However, the limited sample size precluded statistical significance. Both findings suggest ACA is related to patients with typical features of BWS, whereas patients with atypical forms of BWS tend to develop ACC. Thus, patients with atypical forms of BWS are at higher risk of malignancy, and the BWSp clinical score cannot correctly identify patients with BWS at high risk of ACC. ACCs were accurately classified using AFIP/Wieneke’s classification, with the exception of cases in which the AFIP/Wieneke score was 2 or lower, suggesting a benign outcome. These findings highlight the complexity of patients with BWS and ACC and asks for improved diagnostic tools and surveillance [46].

### Somatic Variants

#### Loss of Heterozygosity

In addition to germline alterations of chromosome 11p15, somatic alterations are also common. Loss of heterozygosity of chromosome 11p15 is an early molecular hallmark of pediatric ACT and is found in 91% of pediatric ACTs [6]. While there is a loss of the maternal chromosome, tumor cells still possess the paternal chromosome in all cases. *IGF2* is located on chromosome 11p15 and is only paternally expressed, leading to overexpression in all ACTs, due to an imprinting defect [6]. SNP array profiling of 25 pediatric ACTs showed similar results regarding LOH of chromosome 11p15 in 21 tumors, with *IGF2* being differentially expressed [36]. The frequent occurrence of LOH at 11p15 may reflect underlying genomic instability, influenced by p53 dysfunction. Expression of CDK inhibitor p57Kip2, expressed from *CDKN1C*, is reduced in ACTs. As described, LOH of chromosome 11p15 is also seen in BWS, increasing the risk of developing cancer [36, 47, 48]. In addition, patients with ACC and germline *TP53* variants also show LOH of chromosome 17, on which *TP53* is located.

#### ATRX

Somatic variants of *ATRX, CTNNB1,* and *TP53* have been described in ACC. In one-third of ACTs, an *ATRX* variant was found. *ATRX* encodes a protein that executes myriad functions, such as regulating transcription of certain genes, enabling chromosomal stability, and regulating DNA repair. All ACTs with an *ATRX* variant also had a germline *TP53* variant. Telomeric length was increased in tumors with a somatic *ATRX* variant, which is probably due to alternative lengthening of telomeres [6]. Co-occurrence of *TP53* and *ATRX* variants is also seen in sarcomas and gliomas [49]. Three groups of ACTs were defined based on whole-genome sequencing (WGS). Group 1 consisted of tumors carrying both germline *TP53* variants and somatic *ATRX* variants and was associated with reduced event-free survival compared to Groups 2 and 3. Group 2 included patients with germline *TP53* variants but wild-type *ATRX*; most carried the R337H variant and exhibited favorable characteristics, such as young age at diagnosis (median 30 months) and small tumor size. Telomere length in this group was not elongated. Group 3 included ACTs without *TP53* or *ATRX* variants [6].

#### Wnt Signaling/β-Catenin Pathway

The Wnt signaling/β-catenin pathway has been thoroughly studied in different cancers, including ACC. In adult ACC, the Wnt/β-catenin pathway has been proven to be upregulated in several ways, leading to cell proliferation, epithelial to mesenchymal transition, and inhibition of apoptosis [50]. Somatic variants of *CTNNB1, ZNRF3, TP53*, as well as copy number variations of *TERT*, are seen in adult ACC [51, 52]. *ZNRF3* stimulates degradation of the Wnt receptor, inhibiting its ability to stimulate cell proliferation. *TERT* may also be involved in Wnt/β-catenin signaling [51]. The Wnt/β-catenin signaling pathway, therefore, plays an important role in the development of adult ACC. Somatic variants of *CTNNB1*, encoding for β-catenin, were seen in 13 out of 71 pediatric ACTs. No patients were carriers of a germline *TP53* variant [6]. In contrast, another study found *CTNNB1* variants in 4 out of 62 patients, with all patients carrying the R337H germline *TP53* variant. Poor outcome was associated with *CTNNB1* mutations. However, most ACTs showed higher *CTNNB1* mRNA expression and β-catenin staining in the nucleus and cytoplasm, which differs from adults who show nuclear β-catenin only with *CTNNB1* variants. Furthermore, genes inhibiting the Wnt/β-catenin pathway were also found to be less expressed. These findings together suggest a role of the Wnt/β-catenin pathway in pediatric ACT, but its complexity should be further unraveled [53].

#### TP53

In patients without a *TP53* germline variant, different somatic variants have been identified using polymerase chain reaction and multiplex ligation-dependent probe amplification. In 9 of 54 cases, a somatic *TP53* variant was found [55]. Regarding *CTNNB1*, 23 of 53 tumors carried a variant. Four of these tumors were carriers of both a somatic *TP53* and *CTNNB1* variant [55]. In three cases, *ATRX* variants were detected [55]. In all 3 cases, a somatic *TP53* variant was identified [55]. This is in contrast with the higher presence (5.5% vs 32%) of *ATRX* variants in children with germline *TP53* variants, confirming the association between these two genes. Nine out of 40 cases showed abnormalities of chromosome 11p15 in the DNA of blood samples [55]. However, 86% of tumors showed LOH of the maternal chromosome of 11p15, suggesting that LOH of this chromosome is a hallmark in pediatric ACT development, regardless of germline variants of genes on 11p15 [54].

In conclusion, several genetic alterations are involved in pediatric ACC development. Germline variants are studied more frequently compared to somatic variants, but both seem to play an important role, and germline variants often co-occur with somatic variants [6]. Since germline variants are often responsible for the early development of pediatric ACC, genetic screening is recommended. In adults, nuclear expression of p53 has been demonstrated to differentiate between ACAs and ACCs [55]. Furthermore, in another study in adults, seven out of nine ACCs harboring *TP53* variants exhibited positive nuclear p53 immunostaining [56]. In a study with 13 pediatric ACTs, 8 were positive for p53 immunohistochemistry, but the relationship to outcome was not described [57]. Das et al. (2016), using immunohistochemistry, found that p53 was significantly overexpressed in pediatric ACC [27]. Nevertheless, discrepancies are frequently observed between p53 immunohistochemical staining and the presence of *TP53* variants, indicating that the correlation between these parameters is limited [57]. Therefore, p53 immunohistochemistry should be interpreted with caution, and genetic testing on *TP53* is recommended. Knowledge of the molecular landscape also helps define prognosis. Somatic *ATRX* variants are associated with adverse outcomes, while germline *TP53* alterations are associated with better outcomes [32]. Further research is necessary to unravel the molecular landscape of pediatric ACC, which will contribute to future therapy development.

## Prognostic Factors

### Patient Characteristics

Age at diagnosis is an independent prognostic factor for pediatric ACC. ACCs are relatively common in children under the age of 4, who generally have significantly better OS rates than older children. Older children often present with larger tumors, with extension into adjacent tissue and positive margins after adrenalectomy [58]. These findings suggest that pediatric ACC is a biologically distinct disease in younger children compared to older children.

ACC in children under 4 years of age may originate from the fetal zone of the adrenal cortex, which did not undergo apoptosis. During pregnancy, the fetal zone produces high levels of dehydroepiandrosterone(−sulfate) (DHEA(S)) and shows high levels of IGF-2. After birth, the fetal zone normally goes through apoptosis, and the definitive zone gives rise to the zona glomerulosa and zona fasciculata of the adult adrenal cortex. The zona reticularis develops later in childhood, leading to adrenarche and a rise in DHEA(S) levels, which had been decreased during the first years of life [59]. In young children (< 4 years) with ACT, high DHEA(S) levels are measured, as well as IGF-2 overproduction in the tumor, indicating these tumors have developed from the fetal adrenal cortex.

### Tumor Characteristics

Certain tumor characteristics are related to prognosis. Some features overlap with the ones seen in the histopathological scoring systems. There is a significant correlation between tumor size and long-term survival. Tumors above 10 cm in diameter were generally correlated with a poorer prognosis compared to smaller tumors. However, size is not an independent prognostic factor of survival [3, 58, 60]. Interestingly, multiple studies provide 10 cm as a cut-off point, which corresponds with a volume of 524 cm^3^ based on a spherical tumor. This is much higher than the 200 cm^3^ mentioned in the COG staging system, which could lead to incorrect determination of prognosis according to stage. Another study of children who underwent adrenalectomy for localized ACC found that tumors measuring 9 cm or less were significantly associated with higher 5-year overall survival [61]. Although the precise tumor size cut-off for predicting prognosis has not been established, it remains an important factor to consider when assessing patient outcomes. A unifying feature of the various histopathological scoring systems is the inclusion of necrosis, underscoring its importance in distinguishing ACA from ACC. Necrosis is also prognostically significant, being associated with both disease recurrence and mortality [25].

### Biomarkers

As mentioned before, histopathological classification of ACTs as benign or malignant does not directly correspond with clinical outcome. The use of biomarkers can help identify tumors with a clinically poor outcome and thus contribute to the diagnostic process and help estimate prognosis (Fig. 6).

#### Ki67

**Fig. 5:**
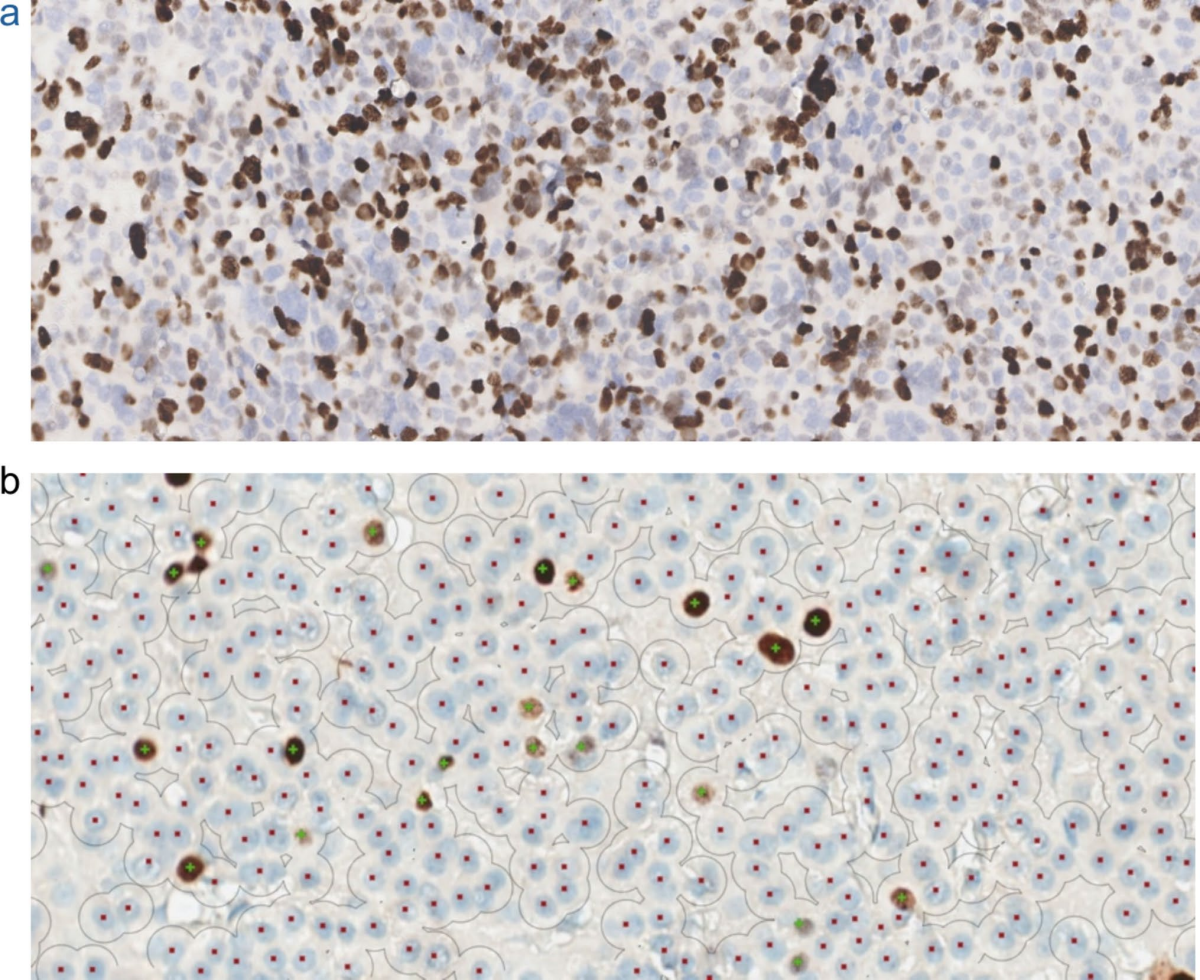
Ki67 immunohistochemistry. **a**) ACC in a 5-year-old boy with Li-Fraumeni Syndrome (Ki67-LI 45%). **b**) Example of Ki67 algorithmic automated counting in a whole slide image of an ACC in a 10-year-old girl (Ki67-LI 4.9%). Green signs indicate positive nuclei; red signs indicate negative nuclei

Ki67 is elevated in most ACCs. Figure 5a shows an example of Ki67 immunohistochemistry. A Ki67-LI above 5% is found in most adult ACCs and should therefore be used to identify malignancy in adults [62]. For pediatric ACC, a Ki67-LI above 10% is found in all ACCs, but a Ki67-LI above 15% is best at predicting outcome. Patients with a Ki67-LI below 15% showed 100% progression-free survival, which led to the inclusion of this threshold in the scoring system by Picard [24]. However, the use of a 15% threshold may fail to identify some malignant tumors. In 2016, a study including 17 pediatric patients found a median Ki67-LI of 2% in ACAs and 12.5% in ACCs [27]. Martins-Filho et al. (2021) compared 146 adult and 44 pediatric ACTs and found a median Ki67-LI in ACA of respectively 0.5% and 7% respectively. In ACC, this percentage was 5% for adults and 26% for children. They compared different cut-off values of Ki67-LI. A Ki67-LI >10% for diagnosing ACC led to 100% sensitivity, but only 51% specificity. Using the same cut-off as Picard et al. (Ki67-LI >15%), they found 89% sensitivity and 77% specificity. Two patients with Ki67-LI <15% did not have progression-free survival. Nevertheless, the cut-off score for Ki67 is affirmed, since high specificity is important to correctly identify malignant ACTs, which is useful when deciding on treatment options [63]. Jangir et al. found the highest specificity and sensitivity (both approximately 80%) at a cut-off score of 18%. However, two patients with a Ki67-LI below 15% did show an unfavorable outcome. Thus, the Ki67-LI remains under evaluation [30]. Preferably, Ki67-LI should be determined using automated analysis in digitalized images (Fig. 5b), as this minimizes the interobserver variation inherent to manual counting [64]. In addition, it should be noted that counting needs to be done in hotspot areas on enough cells (at least 1000 cells).

#### Insulin-Like Growth Factor

Insulin-like growth factor 1 receptor (IGF-1R) is another promising biomarker in separating ACA and ACC. IGF-1R is significantly elevated in pediatric ACC compared to ACA. Insulin-like growth factor 2 (IGF-2) can bind IGF-1R and is differentially expressed in adult ACA and ACC, but not in pediatric cases [65]. In line with these findings, Lira et al. (2016) found overexpression of IGF-2 in pediatric ACTs compared to normal adrenal gland tissue, but no significant association between IGF-2 and survival outcomes. On the other hand, IGF-1R expression was higher in ACTs of patients with recurrence of disease or metastasis. Controversially, IGF-1R was not significantly associated with the survival of patients [66]. IGF-1R is a tyrosine kinase receptor that can induce cell growth, survival, and differentiation by activation of several intracellular pathways [67]. Overexpression of IGF-1R is possibly associated with variants of *TP53*, because wild-type (WT) p53 can repress IGF-1R repression in a completely differentiated cell [68]. Ribeiro et al. (2014) did not find gene amplification of IGF-1R in the pediatric cases they included and did not find microRNA abnormalities associated with IGF-1R overexpression, suggesting these mechanisms are not the cause of high IGF-1R expression [69]. In conclusion, IGF-1R is a potential diagnostic biomarker, but further studies are needed to confirm its significance and prognostic relevance. IGF-2 is elevated in all pediatric ACTs, making it useless in differentiating between benign and malignant.

#### Lamin B1

Overexpression of Lamin B1 can block proteins important for DNA repair, leading to chromosomal instability and a higher chance of the development of different types of cancer [70]. This is also the case for ACC, meaning that overexpression of Lamin B1 leads to a higher mutational rate in tumor cells, stimulating proliferation and invasion. High expression of Lamin B1 was found to be associated with poor prognosis in both adult and pediatric ACC [71]. Future research is needed to verify the role of Lamin B1 as a biomarker in ACCs.

#### Micro RNA

MicroRNA (miRNA) abnormalities have been associated with pediatric ACC as well. MiRNA is important in myriad cell functions, by targeting messenger RNA (mRNA) and regulating gene expression. Four potential biomarkers (hsa-miR-376, hsa-miR-148, hsa-miR-139, and hsa-miR-1305) were able to separate normal adrenal gland tissue from ACT [72]. Seventeen other miRNAs could discriminate between a low- and high-risk group [73]. Patients in the high-risk group tended to have poor OS and event-free survival (EFS), with larger tumors (> 200 cm^3^) and/or metastasis. The study only included two ACAs (according to Weiss criteria) and 35 ACCs, so no conclusions could be drawn about the classification of benign and malignant ACTs with those 17 miRNAs. MiRNAs are promising candidates for diagnostic and prognostic biomarkers, but additional research is required to determine the clinical relevance of each miRNA or a miRNA profile.

#### Spindle Assembly Checkpoint Genes

Several other prognostic factors related to the molecular landscape of ACCs have been identified. One of these factors concerns genes associated with spindle assembly checkpoints (SAC), which can lead to chromosomal instability. These checkpoints are essential to prevent errors during chromosome segregation. Four genes associated with SAC are *AURKA*, *AURKB*, *BUB1,* and *BUBR1*. Expression was measured in ACTs and non-neoplastic adrenals, showing a significantly higher expression of *BUB1* and *BUBR1* in ACTs, but no difference in *AURKA* and *AURKB* expression levels. Overexpression of *BUBR1*, *AURKA,* and *AURKB* was associated with poor outcome, and expression was increased in tumors with a Weiss score of 3 or higher. *AURKA* and *AURKB* overexpression were related to death and unfavorable events. *AURKA* and *AURKB* could therefore be used to determine outcome, and may also be a target for therapy [74].

#### Major Histocompatibility Complex Class II

Major Histocompatibility Complex class II (MHC II) is expressed in children over 4 years on adrenocortical cells in the reticular zone and has been linked to prognosis in pediatric ACT [75]. Low expression of *human leukocyte antigen (HLA)* genes, especially *HLA-DRA*, *HLA-DPA1,* and *HLA-DPB1*, is associated with lower survival [76]. Patients with below-average MHC II expression have a three-year progression-free survival (PFS) rate of 47.1%, while those with above-average expression show a markedly higher PFS rate of 88.2%. Specifically, *HLA-DPA1* was strongly associated with PFS. MHC II expression can also be tested with immunohistochemistry, so MHC II is a potential biomarker useful for diagnosing ACA and ACC [77].

#### DNA Methylation Pattern

In adult ACCs, DNA methylation patterns were analyzed and found useful in differentiating benign and malignant tumors. Different genes playing a role in transcription, cell survival, and the cell cycle are either hyper- or hypomethylated, underlining the effect of epigenetic alterations on tumorigenesis [78]. By analyzing methylation patterns in pediatric ACC with a known outcome, two distinct groups can be identified, with one being associated with a better prognosis than the other. Clay et al. (2019) investigated 48 pediatric ACTs for methylation patterns and divided them into two groups: A1 and A2. The first group (A1), with greater CpG-island methylation, had a higher prevalence of *CTNNB1* variants, which matched β-catenin immunostaining. Group A1 consisted of relatively older children with more advanced disease, and they often received adjuvant therapy. Germline *TP53* variants were enriched in the second group (A2), although not confirmed by immunohistochemistry. OS was significantly higher in the A2 group, since no deaths were registered directly caused by ACT [79]. Bueno et al. (2022) looked at 57 pediatric ACTs, derived from two centers in Southern Brazil. Most patients (86%) were carriers of the R337H variant in *TP53*. They analyzed methylation status and defined two groups of ACT: pACT-1 and pACT-2. Children in the pACT-1 group, having higher methylation in CpG islands, were older, showed symptoms of Cushing’s syndrome more often, and had an overall worse outcome, since more relapses and metastases were seen. In contrast to the first findings, both methylation groups exhibited comparable frequency of *TP53* and *CTNNB1* variants. Disease-free survival (DFS) and OS were drastically lower in the first group, with a 5-year OS of 20%, compared to 98% in the second group. Thus, DNA methylation profiling may serve as an additional tool to histopathologic classification to determine prognosis and therapeutic options [80].

#### Vitamin D3 Receptor

Lastly, the vitamin D3 receptor (VDR) has also been studied in pediatric ACC. In one such work, the vast majority of patients were carriers of the R337H variant in *TP53*. Expression of VDR is reduced in ACT due to hypermethylation of the *VDR* gene. Again, two groups could be created. Children with a lower expression of VDR mRNA tended to be older than 4 years old, present with Cushing’s syndrome and high-stage disease, needing adjuvant chemotherapy. This group therefore had lower disease-free survival (36%) and OS rates (43%), compared to patients with low *VDR* methylation rates and thus higher VDR expression, from whom 85% were alive and free of disease, and 89% and 5% were alive with disease [81].

**Fig. 6:**
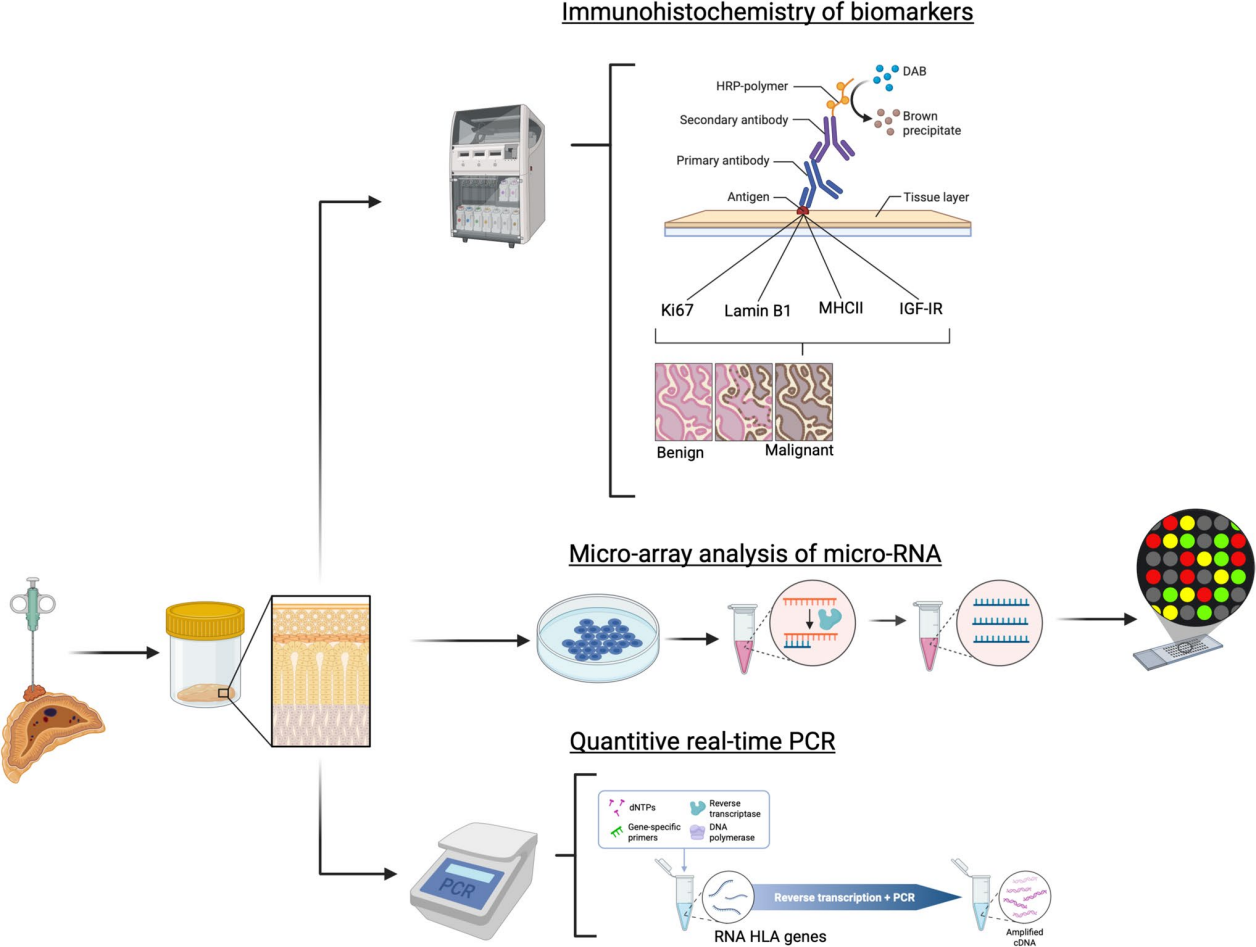
Immunohistochemistry work-up, with possible biomarkers Ki67, Lamin B1, MHC II and IGF-1R, micro-array analysis of micro-RNA, and quantitative real-time PCR of HLA-genes (Created in BioRender. De Krijger, R. (2025) https://BioRender.com/59v5o7h)

Combining these potential diagnostic and prognostic biomarkers (Fig. 6 and Table 3) may help to differentiate between clinically benign and malignant ACTs. Except for automated Ki67-LI detection, these biomarkers have not been implemented in clinical practice. Establishing the significance of these features would offer the chance to create an algorithm allowing for the estimation of prognosis. Age at diagnosis, tumor size, and Ki67-LI are well-established prognostic factors, though cut-off points remain debated. The detection of DNA methylation patterns is a promising new technique for diagnostic and prognostic purposes, so further research in this field could lead to new insights, especially with the use of artificial intelligence. In addition to DNA methylation, other techniques such as whole exome sequencing and mRNA sequencing could be used for clustering. In adult ACCs, molecular clustering effectively stratifies the tumors in prognostic relevant groups [82]. The other factors (IGF-1R, IGF-2, Lamin B1, miRNA, AURK, MHC II and VDR) could be useful but are not yet clinically applicable and would need further validation.

**Table 3:** Summary of potential prognostic factors in pediatric ACC, including survival outcomes for each subgroup. Where possible, survival outcomes are presented as a range, created by taking the lowest and highest percentages reported in referenced studies. OS: overall survival. EFS: event-free survival. PFS: progression-free survival. DFS: disease-free survival

Prognostic factor	Subgroup	Outcome
Age	< 4 years	3-year OS: 86–88%; [58]
		5-year OS: 90.6–100%; [4, 78, 86]
		5-year EFS: 81.2–85.6% [15, 74]
	≥ 4 years	3-year OS: 37–42% [58]
		5-year OS: 21–75% [3, 60, 74]
		5-year EFS: 36.9–59.1% [15, 74]
Tumor size	≤ 10 cm	3-year OS: 76% [58]
		5-year OS: 80–80.9% [3, 60]
	> 10 cm	3-year OS: 42–56% [58]
		5-year OS: 36–46.9% [3, 60]
Tumor weight	<100 g	5-year EFS: 88.3% [74]
		5-year OS: 97.2% [74]
	≥ 100 g and < 200 g	5-year EFS: 36.7%–87.6% [15, 74]
		5-year OS: 48.5% [74]
Tumor volume	< 200 cm^3^	5-year EFS: 84.4% [74]
		5-year OS: 93.5% [74]
	≥ 200 cm^3^	5-year EFS: 35.2% [74]
		5-year OS: 47.6% [74]
Ki67-LI	<15%	OS and PFS: 100% [24]
	≥ 15%	NA
AURKA expression	Low	5-year EFS: 82.6% [74]
	High	5-year EFS: 34.3% [74]
AURKB expression	Low	5-year EFS: 77.5% [74]
	High	5-year EFS: 40% [74]
MHCII expression	Above median	3-year PFS: 88.2% [77]
	Below median	3-year PFS: 47.1% [77]
DNA methylation pattern	A1	OS: 40% [79]
	pACT-1	OS: 20% [80]
	A2	OS: 100% [79]
	pACT-2	OS: 83% [80]
Vitamin D3 receptor methylation	Low VDR methylation	DFS: 84% [81]
		OS: 89% [81]
	High VDR	DFS: 36% [81]
	methylation	OS: 43% [81]

## Future Perspectives

Because of its rarity and heterogeneous nature, treating pediatric ACC remains challenging. Hence, further research is necessary to develop effective and disease-specific therapies in pediatric ACC*.* In vitro models are useful for testing therapeutic approaches, but these are limited for pediatric ACC. NCI-H295R was the first cell line developed from a primary ACC in a 48-year-old woman [83]. In 2013, SJ-ACC3 was established by Pinto et al., the first xenograft of pediatric ACC. The xenograft originated from an 11-year-old boy with an ACC, who was not symptomatic at diagnosis, but laboratory tests showed a functional tumor. The tumor showed several criteria in favor of malignancy, such as high weight, large size, extensive necrosis, high mitotic rate with atypical mitosis and invasion of periadrenal fat and capsular veins. Ki67-LI was approximately 60%, substantiating its malignant potential. The xenograft also contained a germline *TP53* variant and showed overexpression of IGF-2, but no variants of *CTNNB1* were found. Because tumor and xenograft characteristics match that of common pediatric ACC, this xenograft could be useful for drug testing. Several different chemotherapeutics have already been tested, and topotecan was found to be a potential new therapy for pediatric ACC [84]. To date, no other pediatric in vitro models exist, but given the genetic diversity of pediatric ACC, the development of new models is imperative. Organoid models are emerging in cancer research and could be used to develop personalized medicine. Baregamian et al. (2023) developed a 3D organoid model from a patient-derived ACC [85]. However, growing organoids from the adrenal cortex is challenging, and an organoid model for pediatric ACC has not been reported so far.

### Targeted Therapy

Diagnostic biomarkers for pediatric ACC are relevant for therapy guidance and the development of targeted therapy. Systematic NGS analysis in primary or relapse tumors may also inform subsequent targeted therapy. The IGF pathway is important in the development of several malignancies, and overexpression of both IGF-2 and IGF-1R has been reported in pediatric ACC, commonly caused by LOH of chromosome 11p15. OSI-906 (linsitinib) is a potential selective inhibitor of IGF-1R and insulin receptor (IR) autophosphorylation. Lira et al. (2018) demonstrated that OSI-906 decreases cell viability and hormone production in a human adrenocortical cell line (NCI-H295A). However, a plateau was reached above concentrations of 1 μM, and the maximum decrease in cell viability was 18%, suggesting resistance mechanisms play a role and OSI-906 does not solitarily suffice as ACC therapy [66]. Moreover, OSI-906 has not been proven to be effective in a phase 3 study with adults diagnosed with locally advanced or metastatic ACC [86]. Both studies looked at adult ACC, so the therapeutic effects of OSI-906 on pediatric ACC were not determined. Almeida et al. (2008) found a significant increase in IGF-1R expression in pediatric ACC compared to ACA, whereas this difference was not observed in adult ACC [65]. These findings raise the possibility that IGF-R1 inhibition could have increased efficacy in treating pediatric ACC. Lamin B1 was also mentioned as a diagnostic biomarker, and the authors mentioned therapeutic possibilities, but this has not been studied yet. AURKA and AURKB were defined as possible prognostic markers. Inhibition of Aurora kinase by ZM447439 (ZM) was successful in a primary culture of a pediatric ACT carrying the R337H *TP53* variant [74]. Furthermore, another AURKA inhibitor (MLN8237) was tested in several pediatric tumors and showed antitumor activity [87]. The Wnt/β-catenin pathway is another possible target. Nutlin-3a is a promising inhibitor of the growth of adult ACC-cells with a *CTNNB1* variant [88]. The effects of Nutlin-3a should also be tested in pediatric ACCs. The therapeutic effect of immunotherapy has been studied in only a few adult ACC patients. Programmed cell death-1 (PD-1) is an immune checkpoint receptor on T-cells and binding to its ligand PD-L1 on tumor cells, which inhibits the immune response. Several immunotherapies have been developed to target this interaction. For example, pembrolizumab, an anti-PD-1 antibody, has been studied in adult cases twice, but did not show satisfactory response rates (14% and 23%) [89, 90]. Data in the pediatric population are scarce. Jangir et al. reported that all 25 assessed pediatric ACT cases were PD-L1–negative [30]. In contrast, Geoerger et al. observed partial responses to pembrolizumab in two of four pediatric ACC patients [91].

Due to its complexity, treatment of pediatric ACC requires a personalized approach. Targeted therapies may play a role in clinical management, but further research is needed to determine which strategies, including immunotherapy, are effective. As discussed, germline and somatic variants are differentially involved in development of pediatric ACC. consequently, combination therapies are likely to be beneficial. Moreover, the ability to organoids of pediatric ACC in the future could provide an opportunity to test drugs and develop individualized therapy strategies.

## Conclusion

In conclusion, acknowledgment of the different characteristics of pediatric ACC is crucial for determining diagnosis, prognosis, and treatment options. This review outlines our current understanding of the histopathological and molecular classification of pediatric ACC, highlighting its differences from adults. Because of its rarity, collaboration between specialized centers treating children with ACC is important to collect sufficient clinical, histopathological. and molecular data. Ultimately, this will contribute to a better understanding of pediatric ACC, improving survival outcomes for children with this disease.

## Data Availability

No datasets were generated or analyzed during the current study.
